# Electrophoretic Deposition for Lithium‐Ion Battery Electrode Manufacture

**DOI:** 10.1002/batt.201900017

**Published:** 2019-04-02

**Authors:** Cornel C. Lalau, Chee T. John Low

**Affiliations:** ^1^ Cell Manufacture Scale-up in Electrochemical Engineering Group WMG Energy Innovation Centre University of Warwick Coventry CV4 7AL United Kingdom.

**Keywords:** electrode manufacture, electrophoretic deposition, energy storage, lithium-ion batteries, materials science.

## Abstract

Electrophoretic deposition (EPD) has received increasing attention as an alternative manufacturing approach to slurry casting for the production of battery and supercapacitor electrodes. This process is of relevance for industrial scalability as evidently seen in the current electrophoretic paints industry. Nevertheless, the reported work so far have only concentrated on thin films of electrophoretically deposited electrodes for energy storage. Here, the electrochemical performance of thick films (up to tens of μm) as lithium‐ion battery electrodes produced by EPD is reported. A commercially sourced LiN_1/3_M_1/3_C_1/3_O_2_ (5 to 25 μm particle size) was used in this exemplary investigation. This work shows the production of binder‐free high density active material (>90 %) electrodes. Coin cells were assembled and the battery performance was measured. Tests included: cyclic voltammetry, C‐rate vs capacity, battery cycling and electrochemical impedance spectroscopy. Other investigations also studied: colloidal electrolyte formulation, electrode manufacture, microstructure characterisation and elemental mapping analysis. In short, EPD electrode manufacture can be applied as a platform technology for any battery and supercapacitor material, and the reported manufacturing processes and methodologies represent direct relevance to produce energy storage electrodes useful to practical applications.

## Graphical abstract

**Electrode coating in the enery storage account**: Electrophoretic deposition can be applied for electrode manufacture as a platform technology for any battery and supercapacitor materials, producing more energy dense and/or power dense electrodes that are difficult to fabricate using conventional slurry casting approaches.

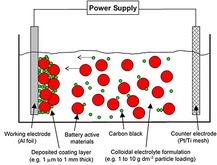

## 1 Introduction

There have been little advances made in the production processes for battery and supercapacitor electrode manufacture, with slurry casting is used for at least the last 30 years. In slurry casting, three types of materials (i. e., electrochemically active materials, carbon‐based electrical conductivity enhancers and polymeric binders) are blended together in an aqueous or non‐aqueous media, and then the resulting paste‐like slurry is physically casted onto current collector through slot‐die or doctor blade. The success of electrode production and the quality of electrode performance are strongly dependent on rheological properties of the slurry, determined by materials faction within the slurry together with drying and calendaring. A recent survey on electrode production, specifically highlighting the challenges to scale‐up lab research to industrial electrode production, is available.^1^ While slurry casting is scalable and robust, new manufacturing advances are needed to produce next generation electrodes when extra performance or functionality is required. While approaches such as sintering, extrusion, and freezing have unlocked extreme values of capacity and rate capability, they generally involved arduous complex processing and poorly suited to produce electrodes at scale suitable for industrial applications.

In the academic science base, the recent years are seeing increasing interests to deploy electrophoretic deposition (EPD) as an alternative manufacturing approach to slurry casting for the production of battery and supercapacitor electrodes.^2^, ^3^

Examples of battery materials studied, Lithium‐ion specifically, include LFP,^4^ LTO,^5^ LMP,^6^ LCO,^7^ LMO^8^ and Si.^9^ A recent comprehensive review on EPD investigations in the fields of battery, supercapacitor and solid oxide fuel cell is available.^10^ It is noted that these published studies reported thin films of coating layers, typically several μm in film thickness. In EPD technology, the mass loading of deposited materials and film thickness can be readily controlled by varying the applied voltage, colloidal electrolyte composition and deposition time. Some reports have introduced modifications to manipulate packing density by EPD and then improve electrochemical properties.^11^ Other authors have reported inter‐particle connectivity by compressing EPD electrode,^12^ formation of sandwich‐like layered structure by controlling EPD electrolyte recipes^13^ and cycling performance improvement by annealing EPD electrode.^14^ While these early studies are useful for identifying the potential of EPD for energy storage electrode manufacture, we are still some ways off to producing practical electrodes which demand thicker films of coating layers to provide useful capacity for actual applications.

Electrophoretic deposition is already a proven industrial process for the production of surface coatings, notably in the electrophoretic paints industry since the 1970s. In its simplest form, EPD exploits the direct interaction of charged materials (in the form of solid particles) with an electric field. The electric field moves charge materials to a deposition substrate, then the materials are deposited onto it building up of a film of coating layer. The high level of digital automation, low levels of pollution and ease of scalability are advantageous that have led to the deposition of solid particles of paints onto car bodies on an industrial scale. EPD manufacturing technology continues to offer a wealth of possibilities to produce coatings with controlled properties (e. g., thin or thick, flat or rough, compact or porous, mono or graded layers) depending on the processing conditions. Although our comprehensive understanding of the underpinning mechanisms and fundamental electrochemical engineering aspects are far from complete, this has clearly not prevented the use of this highly versatile and robust EPD manufacturing technology on an industrial scale.

Here, we address the above battery challenge by performing a systematic investigation of EPD electrode manufacture for Lithium‐ion battery. Initially, the formulation of colloidal electrolytes that offers successful electrode manufacture is studied. The aim is to understand the changes in selected deposition parameters and establish basic design rules to produce electrodes. Key investigations include: (a) optimizing materials fraction; (b) exploring deposition parameters; and (c) characterizing deposited microstructures, both surface and cross‐sectional views. Then, the electrodes are assembled into coin cells (vs. Lithium metal) and their electrochemical performance for energy storage are studied: (a) recording cyclic voltammograms, (b) measuring C‐rates vs capacity extraction, (c) successive cycling for charge/discharge; and (d) quantifying electrochemical impedance spectroscopy. The evidence provided here is useful to exemplify how EPD electrode manufacture approach can extend the intrinsic electrochemical properties of active materials to be realized more fully, including improved electrode design such as higher density active material electrode that are binder‐less and thick film to give useful capacity for practical applications.

## 2 Results and Discussion

### 2.1 EPD Electrode Manufacture

**Figure 1:**
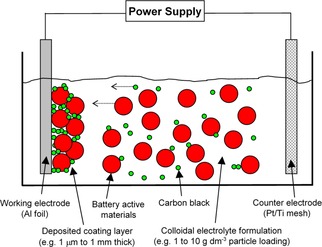
A simplified setup for electrophoretic deposition to manufacture battery electrodes. A controlled migration of battery materials and carbon black particles is induced by the electric field between the electrodes.

A pictorial representation of the electrophoretic deposition (EPD) setup for electrode manufacture is shown in Figure 1. Essential components include a working electrode (i. e., this is the substrate where battery material will be deposited), a counter electrode, a power supply and a colloidal electrolyte (i. e., this is a suspension of battery materials). The process involves three key stages: (1) preparation of colloidal electrolyte, (2) electrophoretic deposition of battery materials onto the working electrode, and finally (3) drying the deposited electrode and use directly as Lithium‐ion battery cathode. Unlike slurry casting approach, no calendaring was performed to densify the deposited electrode and its porous microstructure were resulted directly during deposition.

For successful EPD electrode manufacture, it is critical that the solid materials to be deposited has sufficient surface charge (typically zeta potential±30 mV) so that they can migrate to a deposition surface under the influence of an electric field.^17^, ^18^ It warrants that the colloidal electrolyte has suspension stability so that there is no immediate setting of the solid materials but sufficiently dilute (typically <10 g dm^−3^), offering facile migration of charged materials (which can be of different shapes, sizes and types) to build‐up a film of coating layer on the deposition surface. Charging agents can be added to provide surface charge for solid materials, whilst binders to provide adhesion property between materials‐to‐materials and materials‐to‐deposition surface. Comparing to physical slurry casting approach, the ability to use a controllable electric field to direct the deposition of charged materials which in no doubt drastically increases the technological applicability of EPD to produce highly structured electrode architectures, producing electrodes that are difficult to achieve using viscous slurries that are often prone to suspension instability and fast ageing in conventional battery electrode manufacture technologies.

**Figure 2:**
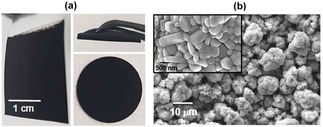
(a) Photographs of electrophoretically deposited electrode. (b) Surface microstructure. Deposition was carried out at a constant voltage of 80 V for 5 min. Colloidal electrolyte contains 4.75 g dm^−3^ NMC (5 to 25 μm), 0.25 g dm^−3^ CB (100 to 300 nm) and 0.1 g dm^−3^ PDDA.

EPD electrode manufacture were started with investigations on active material deposition (5 g dm^−3^ LiN_1/3_M_1/3_C_1/3_O_2_) in the absence of a dispersant and charging agent (0.1 g dm^−3^ PDDA) in the NMP media, resulting in 100 % active material electrode. Although these electrodes have demonstrated adhesion to the Al foil current collector surface, they do not have sufficient electrochemical activity for Lithium‐ion energy storage. This behaviour is likely attributed to the lack of electrical conductivity amongst material‐to‐material and material‐to‐current collector surface. Then, keeping the total materials loading at 5 g dm^−3^, electrical conductivity enhancer carbon black materials were added at 5 wt. % into the NMP media. Good adhesion of the deposited layer on current collector surface was observed using this electrolyte recipe, see Figure 2(a), including physical bending tests and punched out discs for coin cells showed no obvious layer delamination. Surface charge of battery materials was recorded through zeta potential measurement. It was found that negative zeta potential was at around −35 mV in the absence of PDDA, whilst the addition of 0.1 g dm^−3^ PDDA produced+35 mV through a steric stabilization mechanism for successful cathodic deposition. Cathodic EPD was necessary so that Al foil current collector would not undergo anodic dissolution.

Table 1 shows film thickness of EPD electrodes, by varying deposition parameters. A constant voltage at 80 V was used in these experiments, and the recorded current density was around 0.5 mA cm^−2^ to 1.0 mA cm^−2^. It was also observed that the film thickness was readily increased by depositing for a longer duration and by using a higher loading concentration of battery materials. For a longer deposition duration, the slow‐down in the growth rate of film thickness (μm min^−1^) was observed, which could be ideal for uniform thickness distribution all over an irregular 3D topography. If the deposit is a porous layer film, the voltage drop across the layer will remain very low due to the availability of continued conductance pathways through the open porous architecture.^19^ The literature suggests that if a sufficiently conductive electrolyte was used, the electrical resistance of the porous layer will not limit its growth rate which points to the possibility of an unlimited film thickness.^20^, ^21^ Evidence from other fields shows that porous layer (typically 10 % to 60 % porosity) in excess of mm film thickness can be easily deposited. For example, 316 μm thick layer using Al_2_O_3_ insulator materials and even up to 5 mm thick layer using electrically conductive TiB2 materials. Both thick layers were deposited in <60 seconds.^22^, ^23^

**Table 1:** Deposited layer thickness vs. selected deposition parameters. For varying deposition duration experiments, 5 g dm^−3^ battery materials and 0.1 g dm^−3^ PDDA were used. For varying battery materials experiments, 5 minutes deposition and 0.1 g dm^−3^ PDDA were used. For varying PDDA experiments, 5 g dm^−3^ battery materials and 5 minutes deposition were used.

Selected deposition parameters	Deposited layer thickness [μm]	Deposited layer thickness per unit time [μm min^−1^]
Deposition duration [minutes] 2 5 10 20	24 59 83 98	12.0 11.8 8.3 4.9
Battery materials [g dm^−3^] 0.8 2 8 16	12 30 55 65	2.4 6.0 11.0 13.0
PDDA concentration [g dm^−3^] 0.2 0.3 0.4 0.5	31 38 34 35	6.2 7.6 6.8 7.0

It was observed that the growth rate of film thickness can be increased by having a higher loading concentration of battery materials in the electrolyte recipe, but stirring of the electrolyte was necessary to minimize materials sedimentation. The limiting effect of PDDA concentration on the deposited film thickness was also observed, and its concentration needs to be adjusted to suit the loading concentration of battery materials. It is critical that the colloidal electrolyte recipe is sufficiently stable, offering fast enough deposition while ensuring the deposited film is thick enough to provide useful capacity and has electrochemical activity useful for Lithium‐ion energy storage.

### 2.2 Microstructures

An example of the deposited electrode is shown in Figure 2(a). By physical observation, the surface of current collector appears to be covered by battery materials. No obvious pin‐holes can be seen. Following drying and cutting sequences, the electrode disc continues to show good mechanical integrity and no obvious deposits flaking‐off from the edges.

An extreme bending at 180 degree, using a lab twizzle, shows no obvious crack lines along the bended section and no coating layer delamination (i. e., did not peel off) from the Al foil current collector surface. All these physical observations point towards the suitability of EPD approach to produce mechanically robust films for Lithium‐ion battery electrodes. Figure 2(b) shows top surface microstructure, having micron‐sized agglomerates. The granule NMC, having an average diameter of 10 μm, is composed of primary particles with sizes in the range of 500 nm.

**Figure 3:**
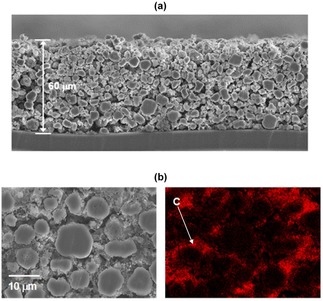
(a) and (b) Cross‐sectional view of the deposited electrode in Figure 2 showing 60 μm film thickness, and (c) the placement of electrical conductivity enhancer carbon black (red color represents the presence of carbon element).

Figure 3(a) shows a cross‐sectional view of an electrophoretically deposited electrode. The film thickness was about 60 μm and mass loading of battery materials was about 15 mg cm^2^; deposition was carried out at a constant voltage 80 V for 5 min in an NMP‐based colloidal electrolyte containing 4.75 g dm^−3^ NMC (5 to 25 μm particle size), 0.25 g dm^−3^ CB (100 to 300 nm particle size) and 0.1 g dm^−3^ PDDA. The electrode shows open pore networks with tortuosity which extends from top to bottom of the film, offering beneficial spacing for Lithium‐ion to access the entirety of the film thickness. This allows, in return, access to all the available capacity of thick film electrodes.^24^, ^25^, ^26^ Clearly in Figure 3(b), elemental analysis for the presence of carbon shows that the smaller particle size of carbon black was infiltrated into the available spacing between the micron‐sized agglomerates. Around 10 wt. % carbon element (detected by Energy‐dispersive X‐ray spectroscopy) was recorded in this electrode.

This strategic placement of carbon black thereby gives the necessary electrical connection at the interfaces of material‐to‐material and material‐to‐current collector surface, allowing the whole electrode to give useful electrochemical activity for energy storage. High density active materials electrode allows the manufacture of both thin electrode for high rate and thick electrode for high capacity, without sacrificing performance by an undesirable quantity of inactive materials. The porous architecture also facilitates improved electrolyte penetration, diffusion and migration, thereby gives enhanced electrochemical performance useful to all power extraction capabilities.

All electrophoretically deposited electrodes were not calendared in this work, but gives a controllable mass loading of battery materials at about 3 mg cm^−2^ to 30 mg cm^−2^. This implies the possibility that EPD approach may simplify electrode manufacture units operation. In all cases, the electrode design and its film thickness must be optimized to give useful combinations of electronic, ionic and interfacial charge transports that maximize the rate at which active materials within the whole electrode can be utilized effectively. Hereafter, the electrochemical performance of electrodes (as in Figure 2) is reported unless otherwise stated.

**Figure 4:**
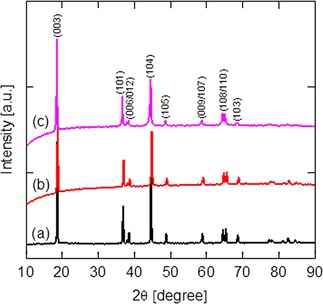
XRD patterns. a) As‐received NMC powder as received from the commercial supplier. b) Electrophoretically deposited electrode film. c) Electrochemically cycled electrode, collected from a lithium‐ion coin cell. For (b) and (c), high active materials density electrode is binder‐less and contains 90 wt. % NMC and 10 wt. % CB.

**Figure 5:**
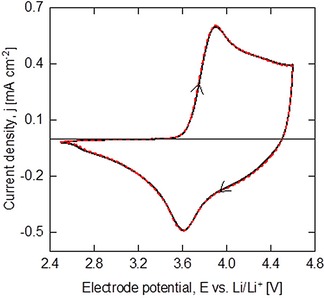
Cyclic voltammogram of an electrophoretically deposited electrode containing 90 wt. % NMC and 10 wt. % CB. Potential scan rate: 0.1 mV s^−1^. Deposition parameters: 80 V for 5 min. Colloidal electrolyte: 4.75 g dm^−3^ NMC, 0.25 g dm^−3^ CB and 0.1 g dm^−3^ PDDA.

Figure 4 shows the recorded XRD patterns namely (a) as‐received powder of NMC, (b) as‐deposited electrode containing 90 wt. % NMC and 10 wt. % CB, and (c) electrode following a successive 50 charge/discharge cycles in a Lithium‐ion coin cell. The XRD pattern for NMC is as expected. This can be indexed to a hexagonal α‐NaFeO_2_ structure with R_3_‐m space group without any impurity,^27^ manifesting crystallized and layered structure.^28^, ^29^ The splitting doublets (006/012) and (108/110) at around 38° and 65° were identified, and the material has a unique 2D tunnel for Lithium‐ion diffusion along a (or b) axis for high capacity and rate performance characteristics.^30^, ^31^, ^32^ For Figure 4(b) and (c), the fluctuation around the baseline at low 2θ angle<20° can be assigned to the existence of amorphous carbon black in the deposited electrode,^33^ but exhibits similar XRD pattern to the as‐received NMC powder. The (003) and (104) diffraction peaks in Figure 5(c) clearly shows a slight shift towards lower 2θ angle suggesting a minor expansion in lattice parameter due to the successive cycling operation.

### 2.3 Electrochemical Cycling Performance

Prior to data collection, two formation cycles were performed at C/10 in the potential range of 2.5 V and 4.5 V vs. Li/Li^+^. The cycles delivered a reversible capacity of 164 mAh g^−1^, recording 87 % coulombic efficiency. Initial irreversible capacity was around 20 mAh g^−1^, as a result of solid electrolyte interface formation at the anode and probably due to an inefficient cathode‐electrolyte interface during the early cycles.^34^, ^35^ Figure 5 shows a typical cyclic voltammogram response of the deposited electrode. Despite the electrode was relatively thick (at around 60 μm film thickness), the clear peaks representing electrochemical redox reactions were recorded.^36^

Comparing with state‐of‐the‐art, slurry casted electrodes tend to bury the surface of active materials, limiting electrolyte access to the available sites for electrochemical reaction and leading to poor rate capabilities. Slurry casted electrodes also contain polymeric binders which can be difficult to de‐convolute the electrochemical properties of active material from cyclic voltammogram response. In Figure 5, the anodic (oxidation) peak at 3.89 V vs. Li/Li^+^ for lithium extraction and a cathodic (reduction) peak at 3.62 V vs. Li/Li^+^ for lithium insertion, showing excellent electrochemical activity at thick film and porous architecture.

Published studies indicate that LiNi_1/3_Co_1/3_Mn_1/3_O_2_ as cathode material, where, upon de‐lithiation, the valences of Ni and Co increase from +2 to+3 and +3 to +4, respectively. The average lithium storage voltage is ca. 3.75 V, as determined by the Ni^2+^/Ni^3+^ and Co^3+^/Co^4+^ redox couples.^37^ The valence state of Mn do not change during charge/discharge processes, but the cations with crystal field stabilization energy at octahedral sites facilitate the overall stability of the material framework and remain electrochemically inactive.^38^

**Figure 6:**
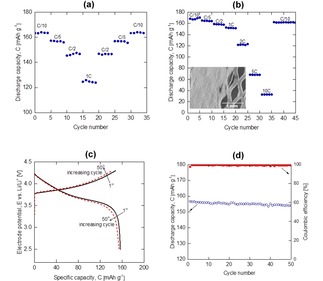
Successive cycling investigations. Rate capability tests on (a) 2D foil and (b) 3D mesh Al current collector. Charging C‐rate was set at C/10 and discharging at various C‐rates. (c) Typical charge/discharge cycles and (d) the extracted parameters from (c).

The extractable capacity of EPD electrodes were investigated under various C‐rates. Figure 6(a) and (b) show the recorded responses of rate capability tests; 2D foil and 3D mesh Al current collectors were used as the deposition substrates respectively. C/10 was used as the charging rate until the electrode potential reached 4.3 V vs. Li/Li^+^ and held until the current reached 0.1 mA before discharging at various C‐rates to 2.5 V vs. Li/Li^+^. A cut‐off at 4.3 V vs Li/Li^+^ was chosen based on the supplier's datasheet. Taking an example in Figure 6(a), discharging the electrode at C/10 delivers around 165 mAh g^−1^ capacity. It was found that the amount of extractable capacity reduces at higher C‐rates, consistent to typical observations for Li‐ion battery materials.^39^, ^40^ Although the discharged capacity at 1 C was only 124 mAh g^−1^, equivalent values were recorded in the reversed C‐rates tests (from 1 C back to C/10). The results clearly showed an efficient capacity performance recovery, suggesting cycling robustness of these EPD electrodes even though they contained a very high active materials density (90 wt. %), binder‐less and thick film (60 μm).

To further advance this finding, battery materials was electrophoretically deposited onto 3D mesh Al current collector (40 % open porous area). Figure 6(b) shows the rate capability response. The inset shows an example of the deposited electrode, showing good coverage of the deposits on mesh strands and the available porous area was filled‐up with the deposits. Over 30 mg cm^2^ mass loading and 150 μm thick film were successfully deposited on 3D mesh; twice as thick as and higher loading than 2D foil as Al current collector. To show mesh coverage, some deposits were scrapped off to reveal the underlining microporous architecture. It was found that discharging at lower rates (C/10 and C/5) of this 3D mesh electrode delivered almost the same capacities as those observed in 2D foil Al current collector. It was only when discharging at higher C‐rates (>C/2), much larger capacities were recorded on 3D mesh than 2D foil. Taking 1 C as an example, about 23 % more capacity was extracted in 3D mesh (152 mAh g^−1^ capacity) compared to 2D foil (124 mAh g^−1^ capacity). Cycling at higher C‐rates (2 C, 5 C and 10 C) that are not designed for this battery material was successful, but sacrificing the amount of extractable capacity. As an example, 10 C discharge had a capacity over 38 mAh g^−1^. Remarkably, the electrode was capable of recovering back to 163 mAh g^−1^ when returning to a lower rate at C/10, again showing cycling robustness of EPD electrode. The excellent rate capability on this granule NMC electrode was probably due to efficient Lithium‐ion diffusion pathway and electrode‐electrolyte contact area through the porous architecture of electrode design.

Galvanostatic charge/discharge cycles were performed, demonstrating the impact of successive cycling on the deposited electrode. Specific capacity of the coin cell was calculated according to the weight of the cathode active materials. Figure 6(c) shows a typical charge and discharge curves, using 2D foil Al current collector. The current at C/5 (30 mA g^−1^) was both charge and discharge rates. Figure 6(d) shows the recorded capacity and coulombic efficiency vs. successive cycles. Coulombic efficiency increases immediately from 97.6 % (first cycle) to about 99.3 % (second cycle), which then remains relatively constant over the subsequent cycles. The electrode delivers around 156 mAh g^−1^, showing 98 % capacity retention over 50 cycles. The electrode potentials of the charge and discharge curves slightly changes with successive cycles, showing electrode polarization upon cycling.

### 2.4 Electrochemical Impedance Spectroscopy Analysis

**Figure 7:**
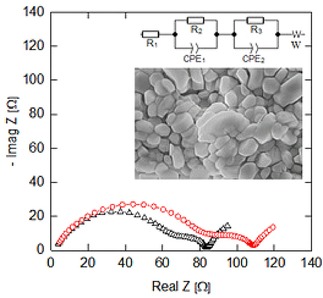
Electrochemical impedance spectroscopy (EIS) response (10 kHz to 10 mHz) of the electrophoretically deposited electrode. ▵ Cycle 5. Cycle 50.

Electrochemical impedance spectroscopy response of the electrophoretically electrodes was carried out at Cycle 5 and 50, being recorded straight after de‐lithiation cycle (open circuit voltage=4.2 V vs. Li/Li^+^) and lithiation cycle (open circuit voltage=3.65 V vs. Li/Li^+^). Exemplary of Nyquist plots are presented in Figure 7, showing semicircles and a slope. The x‐axis intercept of semicircles were used to estimate various resistances, consistent to the approaches in published literature.^41^, ^42^, ^43^

Figure 7 inset is scanning electron microscopy image of the electrode investigated. The semicircles in high/medium frequency region indicated the ohmic resistance (R_1_), solid electrolyte interface resistance (R_2_) and charge and charge transport resistance (R_3_) at the interface between electrodes and electrolyte, while the inclined lines in the low frequency domain represented the Warburg impedance reflecting Lithium‐ion solid‐state diffusion in the electrode materials.^44^, ^45^ The semicircles became larger upon successive cycling, indicating increase in charge transfer and SEI resistances.

In the particular case of Lithium‐ion battery, EIS enables the identification and study ofdifferent processes, including:

- movement of charge carriers through the electrolyte, current collectors and wires (ohmic resistance at frequencies typically above 1 kHz),
- electrochemical double layer and charge transfer reaction at the electrode surfaces (frequencies between 1 Hz and 1 kHz), and
- solid‐state diffusion of lithium‐ions within the bulk of the electrode material (frequencies usually below 1 Hz).

Table 2 compares the magnitude of resistances at de‐lithiated state (battery charged) and lithiated state (battery discharged). It is clear that R_1_ ohmic resistance remains unchanged, but state of charge affected the magnitude of R_2_ solid electrolyte interface resistance and R_3_ charge transfer resistance. Taking an example at Cycle 50, charge transfer resistance increases from 23 Ω (de‐lithiated state) to 137 Ω (lithiated state); whilst solid electrolyte interface resistance increases from 81 Ω (de‐lithiated state) to 344 Ω (lithiated state). All these findings are consistent to observations reported for Li‐ion battery materials in the published literature.

**Table 2:** Values of resistance were extracted from Figure 8. An equivalent circuit model common to Lithium‐ion battery analysis was used. R1 (ohmic resistance), R2 (charge transfer resistance) and R3 (solid electrolyte interface resistance).

EIS resistances	Resistances recorded after de‐lithiation [Ω]	Resistances recorded after lithiation [Ω]
R1 R2 R3	Cycle 5 2 14 66	Cycle 5 2 104 241
R1 R2 R3	Cycle 50 2 23 81	Cycle 50 2 137 344

Lithium‐ion diffusion coefficient (D_Li_^+^) deposited electrode was calculated from Equation (1), using the extracted Warburg impedance coefficients (σ_W_) from Equation (1):^46^, ^47^, ^48^

(1) $${\;\;\;D}_{{Li}^{+}}=\frac{R^{2}T^{2}}{2A^{2}n^{4}F^{4}C^{2}\sigma_{W}^{2}}$$

(2) $$Real\;Z=R+\sigma_{W}\omega^{-1/2}$$

D_Li_^+^ is Lithium‐ion diffusion coefficient (cm^2^ s^−1^), R is gas constant (8.314 J mol^−1^ K^−1^), T is absolute temperature (K), A is electrode area (cm^2^), n is number of electrons involved in the redox process (assuming 1), C is the lithium‐ion concentration (assuming 7.69×10^−3^ mol cm^−3^), F is the Faraday constant (96486 C mol^−1^) and σ_W_ is Warburg impedance coefficients.

**Figure 8:**
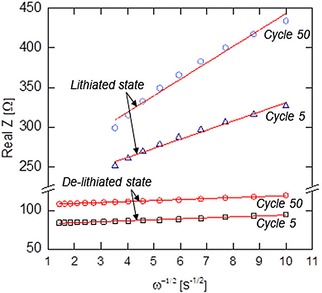
The relationship between Real Z and ω^−1/2^ in the low frequency region, extracted data from EIS Nyquist plots.

Taken from the linear fitting of Real Z vs. ω^−1/2^ (in the low frequency region), Lithium‐ion diffusion coefficients for lithiated electrodes were estimated to be 1.28×10^−14^ cm^2^ s^−1^ (Cycle 5) and 3.95×10^−15^ cm^2^ s^−1^ (Cycle 50) in Figure 8, which is consistent to findings for longer diffusion paths.^49^, ^50^ Based on the data for de‐lithiated electrodes, Lithium‐ion diffusion coefficients were estimated to be around 1.07×10^−12^ cm^2^ s^−1^ (Cycle 5) and 1.10×10^−12^ cm^2^ s^−1^ (Cycle 50). This clearly suggests an efficient cyclic insertion and extraction of lithium‐ion through thick film of the binder‐less, high density active materials electrode.

## 3 Conclusions

Lithium‐ion battery electrodes based on commercial active material Ni_1/3_Co_1/3_Mn_1/3_O_2_ were successfully manufactured by the electrophoretic deposition (EPD) approach. These electrodes contained a high density active material (90 wt. %), and the rest was carbon black as electrical conductivity enhancer material (10 wt. %). Non‐aqueous NMP‐based colloidal electrolytes were formulated using Poly(diallyldimethylammonium chloride) as a dispersant and charging agent, thus enabled cathodic EPD. Using 2D foil Al current collector as the deposition substrate, a film thickness about 60 μm and 15 mg cm^−2^ mass loading were deposited. However, twice as thick and higher mass loading on 3D mesh was achieved than 2D foil. No polymeric binders and post‐electrode calendaring were needed to render the electrode usable for Lithium‐ion energy storage, offering potential to simplify electrode manufacture units operation. Electrode architecture had open pore networks spanning from top to bottom of the thick film, where carbon black (100 to 300 nm particle size) were infiltrated into the available spacing between the agglomerates matrix of Ni_1/3_Co_1/3_Mn_1/3_O_2_ (5 to 25 μm particle size). This work shows a critical evaluation of the important development in EPD for battery electrode research, and advances of this approach vs common slurry casting have on energy storage performance could help to identify its benefits and limitations for future manufacturing research.

The electrode design and its thick film gave useful electronic, ionic and interfacial charge transports. Lithium‐ion diffusion coefficient (1.10×10^−12^ cm^2^ s^−1^), ohmic resistance (2 Ω), charge transfer resistance (23 Ω) and solid electrolyte interface resistance (81 Ω) were recorded; maximizing the rate at which active materials within the electrode was utilized effectively. Electrochemical cycling investigations in coin cell have demonstrated the robustness characteristics of electrophoretically deposited electrodes, including excellent cycling performance useful to a wide range of power extractions (C/10 to 10 C) and a stable 98 % capacity retention (156 mAh g^−1^ @ C/5) over 50 successive charge/discharge cycles. In short, EPD electrode manufacture can be applied as a platform technology for any battery and supercapacitor materials, producing more energy dense and/or power dense electrodes that are difficult to achieve using conventional slurry casting approach.

## Experimental Section

### Materials and Chemicals

Commercially available battery materials were sourced directly from the suppliers, including: active material LiNi_1/3_Co_1/3_Mn_1/3_O_2_, NMC (5 to 25 μm particle size, Targray, Canada) and electrical conductivity enhancer carbon black, CB (100 to 300 nm particle size, 75 m^2^ g^−1^ specific surface area, Alfa Aesar, United Kingdom). An aprotic solvent N‐Methyl‐2‐pyrrolidone, NMP (≥99.5 %, Acros Organics, UK) was selected as the media for EPD experiments, because of its low volatility, high chemical stability, its exceptionally high surface tension (>40 mJ m^−2^) and highly polar nature that ensure a stable suspension of colloids. A related reason is that NMP is recyclable by distillation, biodegradable and is used as the solvent of choice in slurry casting of Lithium‐ion battery electrode. Cationic polyelectrolyte Poly(diallyldimethylammonium chloride), PDDA (20 wt. % in H_2_O, Sigma Aldrich, UK) was used as a dispersant and charging agent. PDDA was chosen because it contains strong cationic and activated adsorbent group radicals which can destabilize and flocculate suspended solids through electro‐neutralization and bridging adsorption.^15^, ^16^ All materials and chemicals were used as received without further processing.

### Electrophoretic Deposition Details

All colloidal electrolyte contains a total of 5 g dm^−3^ materials loading, specifically 95 wt. % active materials (4.75 g dm‐3 NMC) and 5 wt. % inactive materials (0.25 g dm^−3^ CB). These materials were dispersed in NMP solvent firstly, followed by the addition of 0.1 g dm^−3^ PDDA. The media was ultrasonicated and mechanically stirred for 1 hour prior any experiments. A total of 50 mL colloidal electrolyte was used in each experiments. In the EPD beaker cell, a piece of working electrode (4.6 cm^2^ geometric surface area) is placed at a parallel distance (1 cm apart) to a piece of Pt/Ti counter electrode (4.6 cm^2^ geometric surface area). Aluminium 2D foil (15 μm thick; 4.2 mg cm^−2^ mass density) and 3D mesh (25 μm thick; 4 mg cm^−2^ mass density; 40 % porous area) were used as working electrode. Prior to any experiments, Al was ultrasonically cleaned in isopropanol for 30 seconds then washed with deionised water and dried. Cathodic EPD experiments were carried out using a constant voltage (80 V) at room temperature (25 °C), and the colloidal electrolyte was stirred (100 rpm) by a magnetic stirrer bar. Only one side of the Al foil was deposited with NMC/CB; the other side was masked by an insulating tape. The resulted electrodes were left to dry in an air‐filled oven at 80 °C for 12 hours, then transferred to a vacuum oven and stored overnight at 50 °C.

### Electrochemical Performance Analysis

Electrochemical performance was studied in a coin cell setup (CR2032), assembled in an Argon‐filled glovebox (H_2_O<0.1 ppm, O_2_<0.1 ppm). Lithium metal was used as counter/reference electrode, Celgard 2325 microporous membrane as battery separator, and non‐aqueous electrolyte contains 1M LiPF_6_ in ethylene carbonate: dimethyl carbonate (7 : 3 mixture by volume)+1 wt. % vinylene carbonate. All electrochemical measurements were performed using a BT‐Lab system (BioLogic Science). All tests were carried out in a climate chamber at a controlled temperature of 25 °C. Cyclic voltammogram was measured at 0.1 mV s^−1^ from 2.5 V and 4.3 V vs. Li/Li^+^. Charge/discharge cycles were performed at the current density 15 mA g^−1^ to 1500 mA g^−1^ (0.1 C to 10 C rate, 1 C=150 mA g^−1^). Charging cut‐off potential was kept until the charging current decreases to less than C/20, then discharged to 2.5 V vs. Li/Li^+^. For C‐rate vs capacity extraction studies, charging rate was C/10 and discharging at various rates. Over 50 cycles were recorded to show electrochemical cycling robustness of EPD electrodes, as compare to <10 cycles in many published papers which only show the early stages of performance characteristics, but much longer cycling in excess of 1000 s cycles would further quantify the aging behaviour which would be an important topic for real‐world device applications.

Electrochemical Impedance Spectroscopy tests were performed (100 kHz to 10 mHz) using 10 mV AC voltage amplitude. Theoretical capacity of LiN_1/3_M_1/3_C_1/3_O_2_ was assumed to be 278 mAh g^−1^ (molar mass=96.46 g mol^−1^ and electron transfer=1), while the practical capacity delivers 165 mAh g^−1^ when cycled at C/30 in the voltage range of 2.5 V and 4.3 V vs. Li/Li^+^. The practical capacity is lower than theoretical capacity is that not all Li‐ion can be remove from the lattice of the host material. The rest of Li‐ion can be remove above the cut‐off voltage.

### Other Quantitative Characterizations

Zeta potential of colloidal electrolytes were measured using a Malvern Zetasizer Nano ZS (Malvern Instruments). Surface and cross‐sectional views of the microstructures were imaged by a scanning electron microscopy (Carl Zeiss Gemini), supported by an Energy Dispersive X‐ray Spectroscopy (Oxford AZtec). Cross‐sections were prepared by an ion‐milling machine (Hitachi IM4000Plus Ion Milling). Crystal structures were determined by X‐ray diffraction (PANalytical Aeris). Film thickness of the deposited coating layer was measured using a digital thickness gauge from Mitutoyo with a resolution of 1 μm. The amount of materials deposited on the Al current collector was measured using a SE2 ultra‐microbalance from Sartorius with a resolution of 0.1 μg. All equipment and measurement setup were used according to guidelines provided by the suppliers.

Please note: Minor changes have been made to the Acknowledgements section of this manuscript since its publication in *Batteries & Supercaps* Volume 2, Issue 6. The Editor.

## Conflict of interest

The authors declare no conflict of interest.
